# Inter-rater and Intra-rater Reliability of the Chinese Version of the Action Research Arm Test in People With Stroke

**DOI:** 10.3389/fneur.2019.00540

**Published:** 2019-05-29

**Authors:** Jiang-Li Zhao, Pei-Ming Chen, Tao Zhang, Hai Li, Qiang Lin, Yu-Rong Mao, Dong-Feng Huang

**Affiliations:** ^1^Department of Rehabilitation Medicine, The First Affiliated Hospital, Sun Yat-sen University, Guangzhou, China; ^2^Department of Rehabilitation Sciences, The Hong Kong Polytechnic University, Hong Kong, China; ^3^Rehabilitation Department, Shenzhen Hospital, Southern Medical University, Shenzhen, China; ^4^Department of Rehabilitation Medicine, The Fifth Affiliated Hospital of Guangzhou Medical University, Guangzhou, China; ^5^Department of Rehabilitation Medicine, The Seventh Affiliated Hospital, Sun Yat-sen University, Shenzhen, China

**Keywords:** stroke, rehabilitation, upper extremity, action research arm test, reliability, Chinese

## Abstract

**Purpose:** To detect the inter-rater and intra-rater reliability of the Chinese version of the Action Research Arm Test (C-ARAT) in patients recovering from a first stroke.

**Methods:** Fifty-five participants (45 men and 10 women) with a mean age of 58.67 ± 12.45 (range: 22–80) years and a mean post-stroke interval of 6.47 ± 12.00 (0.5–80) months were enrolled in this study. To determine the inter-rater reliability, the C-ARAT was administered to each participant by two raters (A and B) with varying levels of experience within 1 day. To determine intra-rater reliability, rater A re-administered the C-ARAT to 33 of the 55 participants on the second day. Intra-class correlation coefficients (ICCs) and Bland–Altman plots were used to analyse the inter-rater and intra-rater reliability.

**Results:** Regarding inter-rater reliability, the total, grasping, gripping, pinching, and gross movement scores received respective ICCs of 0.998, 0.997, 0.995, 0.997, and 0.960 (all *p* < 0.001), indicating excellent inter-rater reliability in stroke patients. Regarding intra-rater reliability, the corresponding ICCs were 0.987, 0.980, 0.975, 0.944, and 0.954 (all *p* < 0.001), again indicating excellent intra-rater reliability. The Bland–Altman plots yielded a mean difference of 0.15 with 95% limits of agreement (95%LOA) ranging from −2.16 to 2.46 for the inter-rater measurements and a mean difference of −1.06 with 95%LOA ranging from −6.43 to 4.31 for the intra-rater measurement. The C-ARAT thus appeared to be a stable scoring method.

**Conclusions:** The C-ARAT yielded excellent intra-rater and inter-rater reliability for evaluating the paretic upper extremities of stroke patients. Therefore, our results supported the use of the C-ARAT in this population.

## Introduction

Many stroke survivors experience motor deficits (1), particularly in the upper extremities (UEs) (2). These impairments limit the ability of a stroke survivor to perform the activities of daily living (3). Approximately 85% of acute-stage and 55–75% of chronic-stage stroke patients exhibit UE impairment and dysfunction (4). These impairments directly affect the quality of life not only of the individual, but also their family members. This effect is especially pronounced for those middle-aged patients who are the main source of financial support for an entire family (5). Accordingly, efforts enabling stroke survivors to regain their UE function to the greatest extent possible are crucial. Moreover, the efficiency of a rehabilitation strategy relies on an optimal evaluation method that can accurately determine a patient's diagnosis.

The Action Research Arm Test (ARAT) was developed in 1981 by Lyle as a clinical tool for evaluating UE function and dexterity after stroke (6). The ARAT measures the functions of both the arm and hand during various tasks, with particular attention to the fine motor function of the hand. This performance measure comprises tasks similar to necessary daily activities and can be administered quickly and easily. The original ARAT includes 19 items categorized into four subtests (grasping, gripping, pinching, and gross movement), as well as a standardized test kit (Sahlgrenska University Hospital, Gothenburg, Sweden) (7). During this test, function is assessed unilaterally, beginning with the unaffected UE. The scores of each item are then summed to calculate a total score for each side, with a possible range of 0–57 points.

Several studies have demonstrated the good psychometric properties of the ARAT (7–13). Accordingly, this measure has been used widely in clinical and research settings (14–16), and has been applied in ~17% of studies (17). Regrettably, the original version of the ARAT has rarely been used clinically in China. Rather, the UE-Fugl–Meyer Assessment (FMA) is the most commonly used clinical measure for evaluating UE function in stroke patients. Although the UE-FMA assesses the impairment level, it does not adequately assess the level of an activity limitation or the fine motor functions of the hands and fingers. Accordingly, this test is not useful for formulating rehabilitation strategies or determining the effects of such strategies on the recovery of hand function in stroke patients. We therefore translated the original ARAT and its manual into Chinese and explored the internal consistency and concurrent validity of the C-ARAT in our previous study (18). To ensure the robustness and generalizability of this measure, however, its performance must be determined in three main domains of instrument quality: reliability, validity, and responsiveness (19). To our knowledge, no previous study has been conducted to detect the reliability of the C-ARAT. Therefore, this study aimed to investigate the inter-rater and intra-rater reliability of the C-ARAT in patients recovering from a first stroke in China.

## Materials and Methods

### Translation

The original ARAT and its manual were translated from English into Chinese by an expert group using a forward–backward procedure. The translation protocol was published previously (18).

### Subjects

According to previous studies (7, 10, 11, 20), a sample size of 18–35 patients with stroke would be sufficient to calculate the intra-rater and inter-rater reliability of the ARAT. To increase the power of this study, we applied a more conservative sample size. and included 55 inpatients with stroke at the Department of Rehabilitation Medicine of the First Affiliated Hospital, Sun Yat-sen University, China between August 2014 and December 2018. The inclusion criteria were as follows: (1) occurrence of a first stroke with unilateral hemiparetic lesions confirmed by magnetic resonance imaging or computed tomography; (2) an elapsed interval of >6 days after stroke; (3) age of 18–80 years; (4) Brunnstrom motor recovery stage II or higher; (5) Modified Ashworth Scale score ≤ 2; (6) ability to maintain a seated position for >30 min; (7) no severe deficits in communication, memory or understanding and the ability to follow the raters' commands [e.g., Mini Mental State Examination score ≥22] and (8) no other medical, cardiovascular or orthopedic conditions or significant peripheral neuropathy in the UEs. The participants' demographic and major comorbidity data were collected from medical records. The demographic information is presented in Table 1. This study was approved by the Human Subjects Ethics Subcommittee of the First Affiliated Hospital, Sun Yat-sen University. Informed written consent was obtained from all participants before the assessment.

**Table 1:** Characteristics of the study participants (*n* = 55).

**Variable**	**Inter-rater study sample *n* = 55**	**Intra-rater study sample *n* = 33**
	**Values**	**Values**
Age (years)	58.67 ± 12.45 (22–80)	57.70 ± 10.26 (33–78)
Onset (months)	6.47 ± 12.00 (0.5–80)	8.76 ± 14.86 (0.5–80)
Mini mental state examination	26.30 ± 2.86 (22–30)	27.18 ±1.93 (22–30)
**Sex**		
Male (%)	45 (81.82)	29 (87.88)
Female (%)	10 (18.18)	4 (12.12)
**Brunnstrom stage**		
Proximal UE	3.65 ± 1.16 (2–6)	3.58 ± 1.20 (2–6)
Distal UE	3.89 ± 1.27 (2–6)	3.91 ± 1.26 (2–6)
**Stroke type**		
Ischemic (%)	47 (85.45)	29 (87.88)
Hemorrhagic (%)	8 (14.55)	4 (12.12)
**Affected side**		
Right (%)	29 (52.73)	15 (45.45)
Left (%)	26 (47.27)	18 (54.55)
**Dominance**		
Right (%)	55 (100)	33 (100)
Dominant side affected (%)	29 (52.73)	15 (45.45)
Mild problem on speech (%)	20 (36.36)	13 (39.39)

*Values were mean ± SD (range) or n (%)*.

### Procedure

Participants were recruited from the Department of Rehabilitation Medicine of the First Affiliated Hospital, Sun Yat-sen University (*n* = 55) between August 2014 and December 2018. To determine inter-rater reliability, two therapists (raters A and B) who were familiar with the ARAT and could properly administer the measurements according to the guidelines applied the C-ARAT to all participants. Rater A, a physiotherapist with more than 9 years of clinical experience in stroke rehabilitation, received thorough training in the proper administration of the C-ARAT over a period of ~6 months. By contrast, rater B had 1 year of clinical experience in stroke rehabilitation and participated in self-directed training on the clinical application of the C-ARAT ~2 months before the study. Both raters applied the test to each participant in a random order within 1 day and were blind to each other's assessment results during the study period. To assess intra-rater reliability, rater A re-applied the C-ARAT to 33 of the original 55 participants on the second day. The remaining 22 participants were either unable or unwilling to participate in a third round of testing within a 2-day period (this period was established to minimize the possible effect of spontaneous recovery) (8). The C-ARAT was administered in a quiet room. A sufficient rest period was provided during the assessment to eliminate the influence of fatigue on the results.

### Statistical Analysis

#### Participants

The participants' demographic and clinical characteristics were analyzed using descriptive statistics. The results were presented as mean ± SD (range) or n (%).

#### Reliability

Reliability is defined as the degree of similarity between the values obtained by the same rater at different times (i.e., intra-rater) or by different raters at the same time (i.e., inter-rater) (19, 21). In this study, the inter-rater and intra-rater reliability were analyzed using two methods: intra-class correlation coefficients (ICCs) and Bland–Altman plots. The ICCs were used to examine the correlations between repeated measurements obtained from the same patient by different raters or by the same rater at different times. This coefficient indicates the measurement error and agreement as the relationship between the true and observed variances (8). ICC indicates how well the measurement tool can tell subjects apart despite measurement error (22). A high ICC signifies that the measurement tool can effectively grade functional severity after stroke (22–26). The ICC can be calculated by the formula provided below (27):

$$\begin{array}{l} ICC=\;\frac{\sigma_{b}^{2}}{\sigma_{b\;}^{2}\pm \;\sigma_{w}^{2}} \end{array}$$

Where $\sigma_{b}^{2}$ represented the between-group variance of the two successive sets of measurement, and the $\sigma_{w}^{2}$ represented the within-group variance. In order to minimize the effect of spontaneous recovery and learning effect to the result, we used ICC Model 3 (two-way mixed effects, consistency, single-rater and two-way mixed effects, consistency, multiple raters) to quantify the degrees of intra-rater and inter-rater reliability, respectively (28). According to a previous study (29), ICC values of <0.5, 0.5–0.75, 0.75–0.9, and >0.9 are considered to indicate poor, moderate, good and excellent reliability, respectively. Additionally, a Bland–Altman plot was used to compare the mean differences and 95% limits of agreement (LOA) for the total C-ARAT score and thus yield a more detailed analysis of the differences in scores calculated by different raters or by the same rater at different times. Having a similar concept with the minimal detectable change (30, 31), LOA provides the threshold to discriminate real biological change (i.e., the spontaneous recovery of motor impairment) from measurement noise (22). A narrow LOA signifies that the measurement tool can detect more subtle biological change occurring over time or with an intervention (30, 31). The mean difference was calculated as the average of the difference between the two assessments of each subject. The upper and lower boundaries of the 95%LOA were calculated as the mean values from the two assessment sessions ± a SD of 1.96 (32). Here, a smaller mean difference and 95%LOA indicated better agreement.

SPSS version 20.0 (IBM, Inc., Armonk, NY, USA) was used to conduct all statistical analyses and generate the Bland–Altman plots. All tests were two-tailed, and the level of significance was set at a *P*-value <0.05.

## Results

### Demographics

Data were collected from 55 participants (45 men, 10 women) recovering from a first stroke (ischemic, *n* = 47; haemorrhagic, *n* = 8). The mean age of the participants was 58.67 ± 12.45 years (range: 22–80 years). The mean post-stroke duration was 6.47 ± 12.00 months (range: 0.5–80 months). The right side was affected in ~53% of the participants. Details about the 55 participants are provided in Table 1. The C-ARAT total and subscale performance scores obtained by the different raters are summarized in Table 2. The data from the re-evaluations of 33 participants by rater A at different times were pooled to calculate the intra-rater reliability. The details of these 33 participants are also provided in Table 1. The C-ARAT total and subscales performance scores of 33 participants are summarized in Table 3.

**Table 2:** C-ARAT inter-rater reliability.

	**Grasp**		**Grip**		**Pinch**		**Gross**		**Total**	
**Rater**	**A**	**B**	**A**	**B**	**A**	**B**	**A**	**B**	**A**	**B**
Mean	9.91	9.96	6.78	6.75	6.73	6.80	6.18	6.25	29.62	29.76
SD	6.36	6.40	3.74	3.77	6.94	6.90	2.12	2.15	18.10	18.18
Range	0–18	0–18	0–12	0–12	0–18	0–18	3–9	3–9	3–57	3–57
ICC	0.997^a^^**^		0.995^a^^**^		0.997^a^^**^		0.960^a^^**^		0.998^a^^**^	
95%CI	0.994–0.998		0.991–0.997		0.995–0.998		0.932–0.976		0.996–0.999	

*ICC, intraclass correlation coefficient; CI, confidence interval; A, rater A; B, rater B*.

a Excellent correlation

** p < 0.001.

*P < 0.05 indicates significant correlations*.

**Table 3:** C-ARAT intra-rater reliability.

	**Grasp**		**Grip**		**Pinch**		**Gross**		**Total**	
**Examine**	**A1**	**A2**	**A1**	**A2**	**A1**	**A2**	**A1**	**A2**	**A1**	**A2**
Mean	9.39	9.76	6.24	6.42	5.91	6.45	5.97	5.94	27.52	28.58
SD	5.93	5.82	3.53	3.65	6.33	6.57	1.96	1.89	16.63	16.96
Range	0–18	0–18	0–12	0–12	0–18	0–18	3–9	3–9	3–57	3–57
ICC	0.980^a^^**^		0.975^a^^**^		0.944^a^^**^		0.954^a^^**^		0.987^a^^**^	
95%CI	0.959–0.990		0.949–0.987		0.888–0.971		0.908–0.977		0.973–0.993	

*ICC, intraclass correlation coefficient; CI, confidence interval; A1, the first round of evaluations by rater A; A2, the second round of evaluations by rater A*.

a Excellent correlation.

** p < 0.001

*P < 0.05 indicates significant correlations*.

### Inter-rater Reliability

The data of all 55 participants were pooled to calculate the inter-rater reliability. The ICC for the total score was 0.998, indicating excellent inter-rater reliability. The ICCs for the subscale performance scores ranged from 0.960 to 0.997, again demonstrating excellent inter-rater reliability.

### Intra-rater Reliability

For the pooled assessment of intra-rater reliability, the ICC for the total score was 0.987, indicating excellent intra-rater reliability. The ICCs for the subscale performance scores ranged from 0.944 to 0.980, indicating excellent intra-rater reliability.

#### Bland–Altman plot

**Figure 1:**
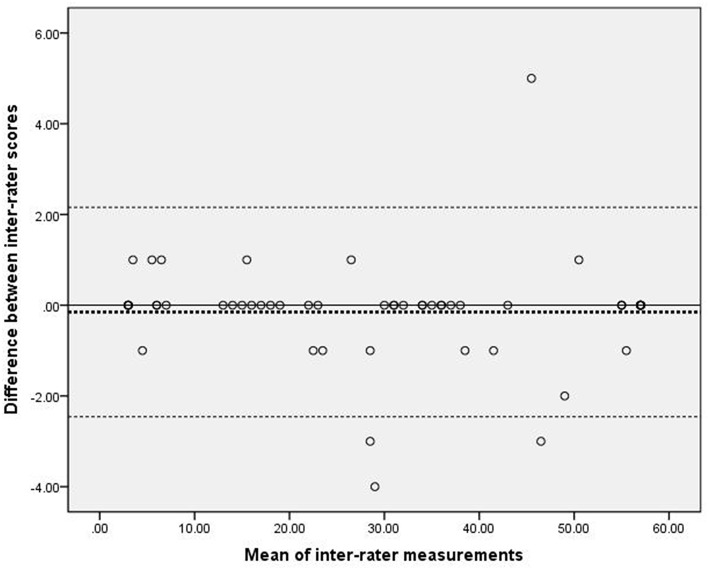
Scatter-plots of the differences between two raters. The dashed bold line represented the mean difference score. The dashed lines represented the limits of agreement (mean ± 1.96 × the standard deviation of the different score).

**Figure 2:**
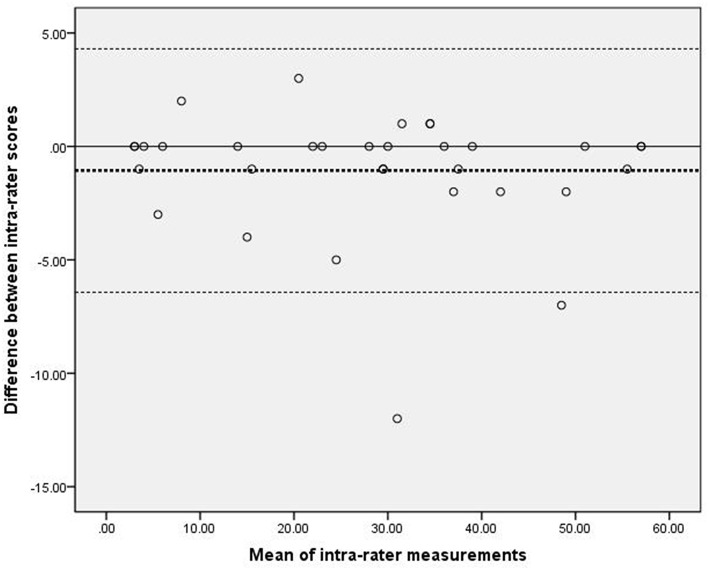
Scatter-plots of the differences between two measurements by the same rater. The dashed bold line represented the mean difference score. The dashed lines represented the limits of agreement (mean ± 1.96 × the standard deviation of the different score).

Figure 1 presents the analysis of inter-rater reliability. The mean difference between the two raters was 0.15, which did not differ significantly from zero. Additionally, the 95% LOA ranged from −2.16 to 2.46, with four outliers. Figure 2 presents the analysis of intra-rater reliability. The mean difference between the two evaluation sessions by rater A was −1.06, which did not differ significantly from zero. The 95% LOA ranged from −6.43 to 4.31, with two outliers.

## Discussion

This was the first study to explore the reliability of the C-ARAT in stroke patients. Our results demonstrated that the total C-ARAT and all four subscales yielded excellent inter-rater and intra-rater reliability. Additionally, a Bland–Altman plot revealed that both the inter-rater and intra-rater evaluations yielded small mean differences and 95%LOA ranges, indicating strong agreement.

Our inter-rater reliability analysis indicated the reliability of both the total C-ARAT and each of the subscales when administered by different raters to the same participants. In other words, different raters achieved consistent results with the C-ARAT, similar to the findings of previous studies (8, 10, 11, 13). Further, our results demonstrated that the different raters not only scored the tests similarly but also tended to assign identical scores to the same individuals. Hsieh reported that the English version of the ARAT was reliable for the assessment of stroke patients (8). In that study, the ARAT yielded good inter-rater reliability when performed by well-trained and experienced (>5 years) therapists. In our study, the raters were trained in different disciplines and had varying levels of experience. Whereas, rater A was well-trained in the ARAT and had considerable experience (>9 years) in stroke rehabilitation, rater B was self-trained and had only 1 year of clinical experience. Despite this considerable difference in clinical experience, the C-ARAT still yielded excellent inter-rater reliability. This may be attributable to the clear instructions and grading of each assessment item. In other words, raters with different levels of clinical experience would similarly perceive the performance of each subject. Additionally, the excellent inter-rater reliability indicated the good psychometric properties and acceptable translation of the C-ARAT.

Our analysis of intra-rater reliability further demonstrated the good reliability of both the total C-ARAT scale and each of the subscales when administered to the same subject by the same rater at different time points. Again, this finding was consistent with those reported in previous studies (11, 13). In our study, the ICCs of the total and pinch subscale scores were lower than those reported by Yozbatiran (13). In contrast to that earlier study, which included only chronic-stage stroke patients (13), our analysis of intra-rater reliability included 18 (54.5%) participants with a stroke onset within 3 months prior to the study. The stage at which a participant is assessed may affect the timing of their UE recovery, such that participants at an earlier stage may exhibit more rapid changes. Accordingly, chronic-stage stroke patients may be better candidates for reliability testing. Alternatively, the interval between assessments could be reduced. Our observations suggest that the C-ARAT is an extremely reliable measure of UE motor function in stroke individuals when performed by the same rater at different time points. This observation was consistent even when the C-ARAT was applied to acute-stage stroke patients, who are strongly subjected to the effect of spontaneous recovery.

Our inter-rater and intra-rater analyses yielded smaller mean differences and 95%LOA ranges, compared to those reported by Nijland (11), who assessed the reliability of the original version of ARAT. Our findings suggest a high level of inter-rater and intra-rater agreement for the C-ARAT. However, ~7.3% (4 of 55) participants in the inter-rater and ~6.1% (2 of 33) participants in the intra-rater analyses fell outside of the 95% LOA. These rates were slightly higher than those reported by Nijland (1 of 18, ~5.6%) (11). Additionally, our two Bland–Altman plots revealed a greater mean difference and 95% LOA in the intra-rater plot relative to the inter-rater plot, suggesting a lower level of intra-rater agreement than inter-rater agreement. Again, this was inconsistent with Nijland's findings (11). Furthermore, the intra-rater plots suggest a less stable scoring method when compared to the inter-rater plots. Our finding may be attributable to the learning effect. In other words, the participants already had previous experience and two rounds of practice with the C-ARAT when they were tested for the third time within 2 days. The spontaneous recovery effect, described above, may also explain our results. Specifically, the 18 (54.5%) participants with a stroke onset within 3 months may have experienced an improvement in UE function within 2 days. In addition, the LOA of Bland-Altman plot has a similar concept with the minimal detectable change (30), which is used to distinguish whether the change stem from the true biological difference or measurement error (33). In this study, we found that 92.7 to 93.9% points fell into the 95% LOA, which implied that the difference of inter-rater and intra-rater measurement were mainly attributed to the measurement error (22). Only 6 to 7% of the difference may be attributing to the biological change. Thus, there was weak impact of real change coming from the spontaneous recovery in this study. From a different perspective, however, our finding suggests that the C-ARAT may be sensitive to changes in UE function. However, further proof is needed to confirm the sensitivity of the C-ARAT.

This study had some limitations. First, the sample size was modest. Therefore, we were unable to conduct analyses according to the severity or type of stroke, type of intervention or duration of training. Second, the enrolled participants had a wide range of stroke onset intervals. As noted, some patients in the sub-acute phase may have experienced a strong effect of spontaneous recovery. Accordingly, these variances in the outcome measure may have led to differences in performance during the C-ARAT. Nevertheless, the C-ARAT demonstrated an excellent inter-rater and intra-rater reliability. Finally, we only evaluated the intra- and inter-rater reliability of the C-ARAT. Further research should explore the comprehensive psychometric characteristics of the instrument, such as the responsiveness and predictive validity, in stroke survivors at different stages.

## Conclusion

In conclusion, our preliminary evidence indicated the excellent inter-rater and intra-rater reliability of the C-ARAT. Accordingly, the C-ARAT appeared valuable for measuring UE function in Chinese stroke patients.

## Data Availability

The datasets generated for this study are available on request to the corresponding author.

## Ethics Statement

This study was approved by the Human Subjects Ethics Subcommittee of the First Affiliated Hospital, Sun Yat-sen University, China. Informed written consent was obtained from all of the participants before the assessment.

## Author Contributions

D-FH, Y-RM, and J-LZ designed the experiment. D-FH, J-LZ, TZ, QL, and HL translated the ARAT scale and manual. J-LZ, P-MC, and TZ performed the experiment and analyzed the data. J-LZ, P-MC, D-FH, and Y-RM interpreted the results and wrote the manuscript. All authors read and approved the final manuscript.

### Conflict of Interest Statement

The authors declare that the research was conducted in the absence of any commercial or financial relationships that could be construed as a potential conflict of interest.
